# Neonatal microbiome dysbiosis decoded by mNGS: from mechanistic insights to precision interventions

**DOI:** 10.3389/fcimb.2025.1642072

**Published:** 2025-08-18

**Authors:** Fangjun Huang, Jiawen Li, Dengjun Liu, Yuling Li, Jun Tang

**Affiliations:** ^1^ Department of Neonatology, West China Second University Hospital, Sichuan University, Chengdu, Sichuan, China; ^2^ Key Laboratory of Birth Defects and Related Diseases of Women and Children (Sichuan University), Ministry of Education, Chengdu, Sichuan, China

**Keywords:** mNGS, necrotizing enterocolitis, neonatal sepsis, neonatal pneumonia, neonatal meningitis, neonatal jaundice

## Abstract

The neonatal period is a critical stage for microbial colonization and immune system development, with dynamic changes in the microbiome closely linked to the pathogenesis of various diseases. Traditional microbiological testing methods have low sensitivity and time-consuming limitations compared to metagenomic next-generation sequencing (mNGS), which makes it difficult to meet the diagnostic and therapeutic needs of critically ill neonates. mNGS analyzes the total DNA in a sample without bias, allowing comprehensive identification of bacteria, viruses, fungi, and parasites, and resolution of functional genes, providing new avenues for precision diagnosis and treatment of diseases such as neonatal sepsis, necrotizing enterocolitis, neonatal pneumonia, neonatal meningitis, neonatal jaundice, and other diseases. However, challenges remain, including the need to optimize sample processing workflows and develop portable devices to enhance clinical conversion potential. In this review, we summarize the application, efficacy, and limitations of mNGS in neonatal diseases. This approach paves the way for novel avenues in mechanistic research, early diagnosis, and personalized therapy for these conditions.

## 1 Introduction

The neonatal period represents the most vulnerable phase of life and a critical window for microbial colonization and immune system maturation (Hansen et al., 2013). The composition and dynamics of the neonatal microbiome (encompassing bacteria, viruses, fungi, and parasites) profoundly influence health (DiBartolomeo and Claud, 2016). Beyond roles in nutrient metabolism, immune development, and intestinal barrier formation, microbial dysbiosis is intricately associated with the onset and progression of neonatal diseases (Jia and Tong, 2020). Preterm and low-birth-weight infants (Vinturache et al., 2016), with immature immune and metabolic systems, are particularly susceptible to microbiome perturbations, leading to heightened disease risks (Jia and Tong, 2020). Traditional microbial detection methods, like culture-based techniques, widely used clinically, can be limiting: a. only culturable microbes (≤20% of total microbiota) can be detected; b. prolonged processing times (days to weeks) delay critical diagnoses; c. low sensitivity and specificity, especially in low-biomass samples like blood or cerebrospinal fluid, increasing false-negative rates (Lewis et al., 2021). Thus, there is an urgent need for rapid and accurate microbial detection technologies.

Metagenomic next-generation sequencing (mNGS) enables unbiased analysis of the full genetic information of microbial communities allowing not only the identification of bacteria, viruses, fungi and parasites, but also the resolution of functional genes of microorganisms like antibiotic resistance genes and virulence factors (Xiong et al., 2023). A retrospective study that included 1493 mNGS samples (including blood, cerebrospinal fluid, and alveolar lavage fluid) from pediatric patients found a higher rate of mNGS positivity compared to conventional microbiological cultures, especially in patients with sepsis who were younger than 6 years of age or in an immunosuppressed state (Zhu et al., 2023). Another systematic evaluation (*n*=462) comprising 5 studies found that mNGS improved the identification of the etiology of neonatal and pediatric sepsis, particularly in negative cultures and in the identification of abnormal microorganisms (bacteria, viruses, fungi and parasites that are difficult to grow in culture) (Agudelo-Pérez et al., 2023). In addition, mNGS provides a scientific basis for microbiome interventions, like probiotics and fecal microbiota transplantation, and opens new avenues for the prevention and treatment of neonatal diseases (Neumann et al., 2023).

At present, systematic reviews on the application of mNGS in neonatal diseases are still scarce. This manuscript is the first to comprehensively sort out its application in various diseases. Therefore, the aim of this review is to describe the potential applications and challenges of mNGS in neonatal diseases from the perspective of neonatal microbiome influencing the development of diseases, to provide clinicians and researchers with certain references to mNGS.

## 2 Metagenomic next-generation sequencing overview

**Figure 1:**
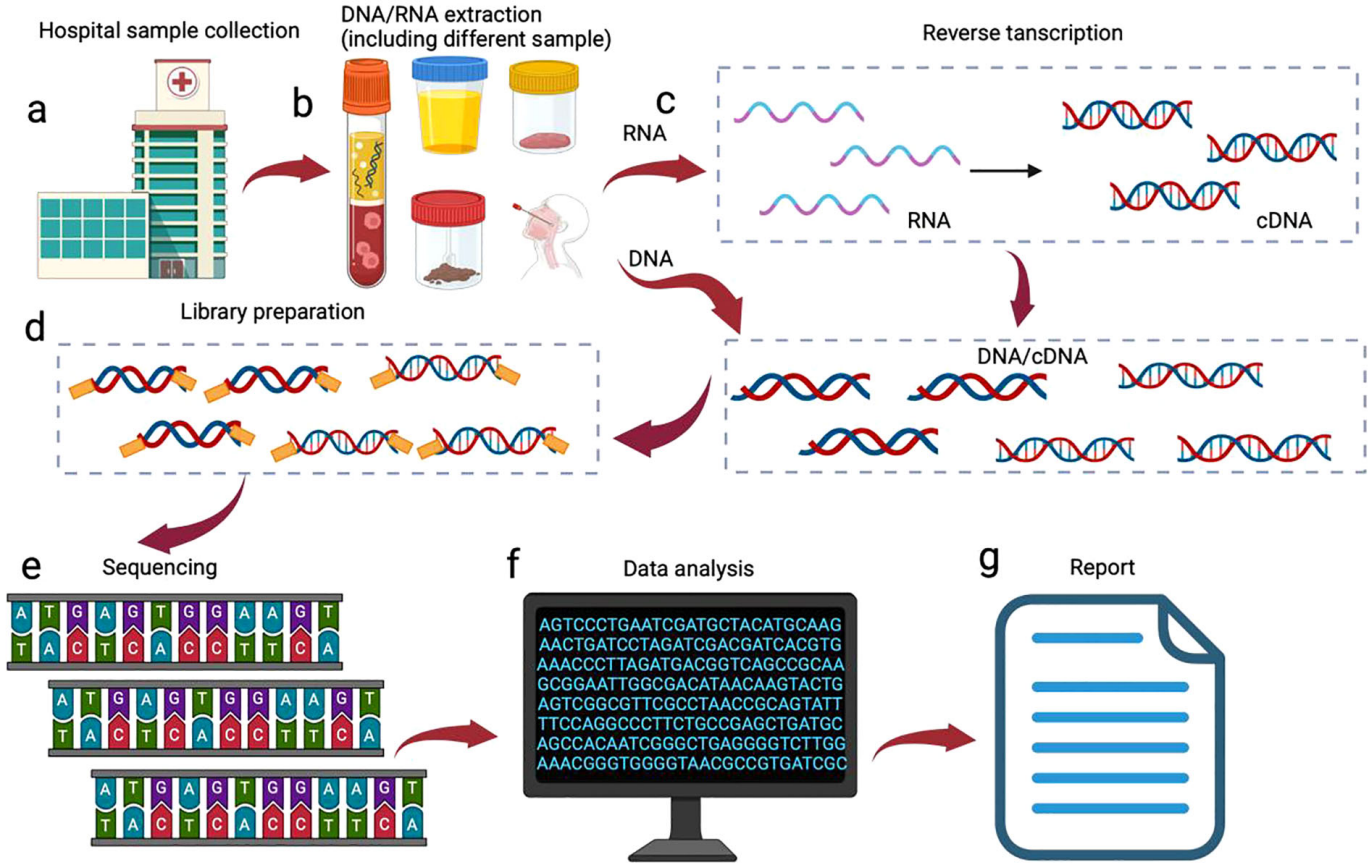
mNGS processes. The mNGS workflow is initiated following clinical confirmation of patient eligibility for testing: **(a)** specimen collection from suspected infection; **(b)** Pathogen-tailored nucleic acid extraction; **(c)** Reverse transcription for RNA sequencing; **(d)** library preparation; **(e)** High-throughput sequencing; **(f)** Bioinformatic processing: human read filtration, microbial alignment to curated databases, and application of validated thresholds; **(g)** Clinical report generation for physician interpretation.

mNGS is a microbiome analysis method based on high-throughput sequencing technology, the core idea of which is to directly extract the total DNA (including microbial and host DNA) from the sample, sequence the DNA fragments by high-throughput sequencing platforms, e.g., Illumina (Illumina; San Diego, California, USA), Pacific Biosciences (PacBio, Menlo Park, California, USA), and Oxford Nanopore Technologies (Oxford Nanopore; Oxford, England, UK). And finally interpret the sequencing data by bioinformatics analytical tools (**Figure 1**).

### 2.1 Sample processing and DNA extraction

The first step in mNGS is sample collection and processing. For neonatal studies, common sample types include faces (gut microbiome), blood (sepsis pathogen detection), respiratory secretions (respiratory infections) and skin swabs (skin microbiome). As neonatal sample sizes are usually small and the percentage of host DNA is high, up to 99% of human DNA in blood samples, experimental procedures need to be optimized during DNA extraction to reduce host DNA interference and increase the yield of microbial DNA. Commonly used methods include lysis of cells using chemicals or osmotic lysates prior to extraction, followed by degradation such as enzymes to release the human genome content, leaving only intact microorganisms (Charalampous et al., 2019) or centrifugation (Chen et al., 2024) to reduce the human nucleic acids for the next step of analysis. To enrich microbial DNA from human oral samples, various host DNA depletion methods show markedly different efficacy: lyPMA treatment (8.53 ± 2.08% human reads), QIAmp kit (29.17 ± 5.04%), and Molysis™ Basic (62.88 ± 3.46%) significantly reduced the proportion of human reads compared to untreated samples (89.29 ± 0.61%). In contrast, the NEBNext^@^ kit (90.83 ± 0.77%) showed no significant effect (Marotz et al., 2018). Other effective approaches include: selective separation of methylated host DNA using methyl-CpG-binding domains (50-fold reduction in human reads) (Feehery et al., 2013) and CRISPR-Cas9-mediated depletion of high-abundance sequences (>99% reduction in mitochondrial rRNA in Eukaryotic samples) (Prezza et al., 2020).

### 2.2 High-throughput sequencing

After the extracted DNA is fragmented, high-throughput sequencing is performed by constructing libraries like short-read-long sequencing libraries from Illumina or long-read-long sequencing libraries from Nanopore). Currently, commonly used sequencing platforms include: 1) Illumina: short-read long sequencing (150–300 *bp*), with high accuracy and high throughput, suitable for large-scale microbiome research; 2) NextSeq (Illumina; San Diego, California, USA): a mainstream sequencing platform for clinical pathogen detection due to its medium throughput and short sequencing time (12–30 hours per run) (Chen et al., 2020); 3) Ion Torrent (Thermo Fisher Scientific; Waltham, Massachusetts, USA): detects H+ released at each dNTP integration, with outputs ranging from ∼50 megabyte to 15 gigabyte per chip, and run times ranging from 2 to 7 hours (Goodwin et al., 2016); 4) Single-molecule real-time sequencing (SMRT; Pacific Biosciences, Menlo Park, California, USA): average read length is 10-20 kilobyte and can achieve 160 gigabyte of data output in 6 hours (Filkins et al., 2020); 5) Nanopore sequencing (Oxford Nanopore Technologies; Oxford, England, UK): produces long reads >200 kilobyte, allows real-time analysis of sequencing data, and can identify pathogens in 6 hours (W. Global tuberculosis report, 2019).

### 2.3 Bioinformatics analysis

Before species annotation and functional analysis, the raw data undergoes quality control, like removal of low-quality reads and host DNA sequences. Commonly used bioinformatics tools include: a. species annotation: Kraken2 (Wood et al., 2019), MetaPhlAn (Blanco-Míguez et al., 2023), Centrifuge (Kim et al., 2016). And other tools classify microorganisms by comparing reference databases, like NCBI (Camacho et al., 2009), Greengenes (DeSantis et al., 2006); b. functional analysis: HUMAnN2 (Franzosa et al., 2018), MG-RAST (Meyer et al., 2008), and other tools predict microbial functional genes by comparing functional databases, like KEGG, COG, to predict the functional genes of microorganisms; c. Resistance gene analysis: databases such as CARD (McArthur et al., 2013) and ARG-ANNOT (Gupta et al., 2014) were used to identify antibiotic resistance genes. The rigorous application of appropriate statistical methodologies constitutes a critical component of bioinformatics workflows, ensuring robust inference and reproducible biological discovery.

## 3 Standardized clinical implementation protocol for mNGS in neonatal diseases

### 3.1 Workflow and clinical application of mNGS

**Figure 2:**
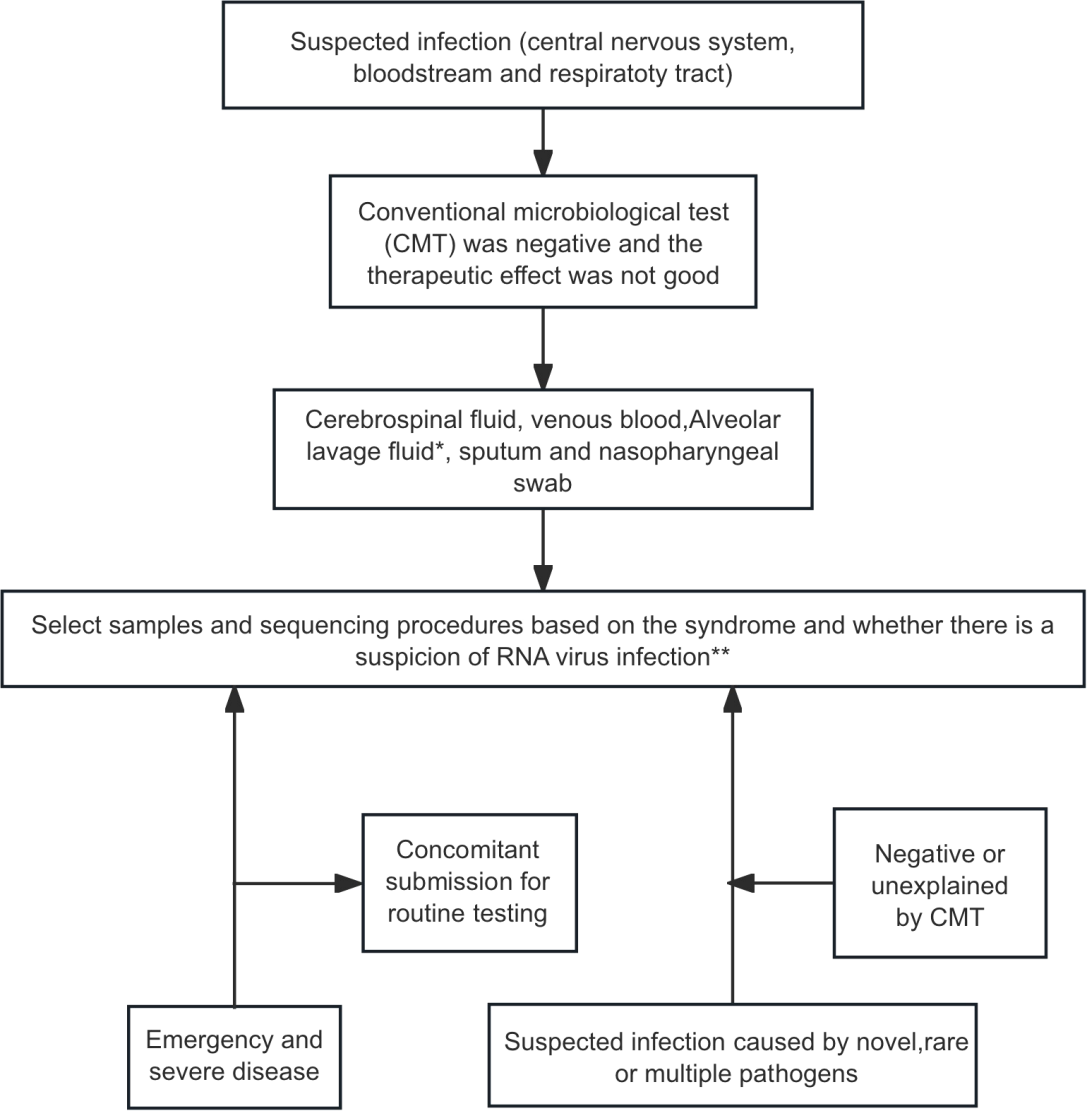
mNGS testing workflow for infection diagnosis in neonatal patients (The Subspecialty Group of Neonatology tSoP, Chinese Medical Association and the Editorial Board, Chinese Journal of Pediatrics, 2022). *Respiratory infection: prioritize alveolar lavage fluid, then sputum or nasopharyngeal swab. **Bloodstream infection, DNA-mNGS first; add RNA-mNGS if RNA virus not excluded. Respiratory infecton, perform both DNA- and RNA-mNGS. Central nervous system infection, DNA-mNGS after viral exclusion; dual DNA/RNA-mNGS when viral etiology suspected, or presentation is indistinct.

The clinial utility of mNGS in neonatal infection management has been validated by multiple international/domestic studies. According to the latest expert consensus, we summarize key elements below in **Figure 2** (The Subspecialty Group of Neonatology tSoP, Chinese Medical Association and the Editorial Board, Chinese Journal of Pediatrics, 2022) and **Table 1** (Infections TFoMN-GSfC, 2019).

**Table 1:** Specimen collection precautions (Infections TFoMN-GSfC, 2019).

Sample type	Volume	Collection tube	Storage conditions	Transport conditions	Turnaround time (TAT)*
Venous Blood	1-3mL (min:0.5mL)	K_2_EDTA/Streck Cell-Free DNA Tubes	6~35°C	6~35°C	24-48h
Cerebrospinal Fluid	1mL (min:600μL)	Sterile Cryovials	**DNA**:-20°C (1 week), -80°C (long-term) **RNA**:-80°C only *Avoid freeze-thaw cycles*	Dry ice	24-48h
Pleural/Peritoneal Fluid	≧5ml				24-48h
Bronchoalveolar Lavage Fluid	≧3ml				48h
Sputum	≧3ml				48h
Throat Swab					24-48h

*: TAT is defined as the interval from sample receipt to report issuance. Significant inter-laboratory variation exists in TAT benchmarks.

### 3.2 Ethical considerations

Given the ethical sensitivity of mNGS testing, clinical implementation is strictly reserved for cases meeting all criteria: 1. Negative conventional cultures with high clinical suspicion of infection, b. Anticipated results carrying critical therapeutic implications. Prior to initiation, attending physicians must provide legal guardians with detailed explanations: a. Purpose, scope, performing laboratory credentials, and costs; b. Potential false-negative/false-positive results due to biological sample variability and technical limitations; c. Performing laboratories’ assumption of full responsibility for diagnostic accuracy and resultant medical consequences. Specimen collection proceeds only after obtaining written informed consent. This protocol strikes a balance between adopting novel diagnostic technologies and protecting patient rights, providing a standardized framework for implementing mNGS in clinical practice (Appendix 1: informed Consent Template).

## 4 Metagenomic next-generation sequencing in the study of neonatal diseases

Current evidence indicates limited mNGS use in neonatal disease. We therefore systematically classified common indications, assessed study quality with the Newcastle-Ottawa Scale, and summarized findings in **Tables 2** (Wells et al., 2000) and **3**.

**Table 2:** The assessment of studies with Newcastle–Ottawa quality assessment scale.

Author	Article type	Selection	Selection of the non -exposed cohort	Ascertainment of exposure	Demonstration that outcome of interest was not present at start of study	Comparability	Outcome	Was follow-up long enough for outcomes to occur	Adequacy of follow up of cohorts	Score
		Representativeness of the exposed cohort				Comparability of cohorts on the basis of the design or analysis	Assessment of outcome			
Yunqian Zhu et al., 2023	retrospective study	★	★	★	★		★			5
Charlotte J Neumann et al., 2023	prospective study	★	★	★	★	★★	★			7
Andrea C Masi et al., 2021	prospective study	★	★	★	★	★★	★			7
Yue Tao et al., 2022	prospective study	★	★	★	★		★			5
Chun-Yan Lou et al., 2024	retrospective study	★	★	★	★		★			5
Mengmeng Ge et al., 2021	prospective study	★	★	★	★		★			5
Shaoming Zhou et al., 2019	prospective study	★	★	★	★		★			5
Juan Ding et al., 2021	prospective study	★	★	★	★★	★	★			7
Hanna Renk et al., 2024	prospective study	★	★	★	★★	★	★			7
Milton T Mogotsi et al., 2024	prospective study	★	★	★	★	★	★			6
Lu Chen et al., 2022	prospective study	★	★	★	★		★			5

**Table 3:** Summary of mNGS applications in neonatal diseases.

Author	Sample size	Study population	Inclusion criteria	Primary outcome	Confounding factors	Sequencing platform	Sequencing depth
Yunqian Zhu et al., 2023	Blood (*n*=615) CSF(*n*=576) BALF(*n*=196) Sputum (*n*=86)	*n*=1473	from patients with suspected or diagnosed sepsis, CNS infection, or lower respiratory tract infection; from patients aged 0 to 18 years; from blood, bronchoalveolar lavage fluid, CSF, or sputum; ested by mNGS and culture with or without other CMT within 3 days using the same sample type.	Microbial composition, abundance	NR	Illumina NextSeq 550	≧ 20M
Charlotte J Neumann et al., 2023	faeces (*n*=399)	*n*=55	preterm infants with BW<1500g; survival in the first three weeks of life	Microbial composition, abundance, and functional traits	Whether bigidobacterium and gentamicin are administered, the feeding plan (formula milk, breast milk, mixed feeding), and the hospital location	Illumina MiSeq	NR
Andrea C Masi et al., 2021	faeces (*n*=644)	*n*=77	born at <32 weeks of gestation	Microbial composition, abundance	GA, whether antibiotics and probiotics are received	Illumina HiSeq X Ten	NR
Yue Tao et al., 2022	Blood (*n*=146) CSF(*n*=69) BALF(*n*=237) Sputum (*n*=62)Tissue (*n*=58)and others*	*n*=519	Suspected infected	Microbial composition, abundance	NR	Illumina Nextseq CN500	NR
Chun-Yan Lou et al., 2024	Blood (*n*=45)	*n*=45	GA <37weeks, survival time >3days; suspected sepsis based on clinical symtoms	Microbial composition, abundance	NR	NR	NR
Mengmeng Ge et al., 2021	Blood(*n*=43) CSF(*n*=101)	*n*=88	Clinically stable newborn with suspected central nervous system infecion	Microbial composition, abundance, and functional traits	NR	Illumina NovaSeq	NR
Shaoming Zhou et al., 2019	faeces (*n*=70)	*n*=70	direct bilirubin in serum >17.1 μmol/L; TBIL<85.5 μmol/L or TBIL > 85.5 μmol/L with DBIL/TBIL >20%; aged 1–5 months old; no recorded use of antibiotics; presenting with skin or sclera jaundice	Microbial composition, abundance	NR	Illumina Hiseq2500	5700-7000x
Juan Ding et al., 2021	faeces (*n*=133)	*n*=25	Exclusively breast fed With a BW of 2500 to 4000 grams GA of 37 to 42 weeks a fifth-minute Apgar score of 8 to 10	Microbial composition, abundance, and functional traits	age, gender, BW, height	Illumina MiSeq	NR
Hanna Renk et al., 2024	faeces (*n*=NR)	*n*=13	Newborns diagnosed with congenital heart disease Full-term newborns Or those schedule to undergo cardiopulmonary bypass surgery within 4 weeks after birth	Microbial composition, abundance	age, gender, BW	Illumina NextSeq	14000x
Milton T Mogotsi et al., 2024	faeces (*n*=68)	*n*=17	at the age of zero during recruitment; who will be residing within the Mangaung metropolitan region during the study; with or without clinical symptoms	Microbial composition, abundance, and functional traits	NR	Illumina MiSeq	NR
Lu Chen et al., 2022	Blood (*n*=153) CSF(*n*=127) BALF(*n*=39) Sputum (*n*=10) Respiratory secretion(*n*=2)	*n*=168	hospitalized from January 1, 2020 to June 30, 2021 suspected of having infections in the central nervous system, bloodstream, respiratory tract, intestinal system or urinary system	Microbial composition, abundance	NR	Illumina NextSeq 550	4000–5000×

*others means hydrothorax(n=17), ascites(n=18), pericardial effusion(n=7), nose/mouth swab (n=5), secreta(n=3), pus(n=13), others(n=5).

CSF, cerebrospinal fluid; BALF, bronchoalveolar lavage fluid; CNS, central nervous system; mNGS, metagenomic next-generation sequencing; CMT, conventional microbial testing; NR, not reported; BW, birth weight; GA, gestational age.

### 4.1 Necrotizing enterocolitis

The gut microbiome of children with NEC shows instability before disease onset and is associated with specific bacterial species. For example, a study (Masi et al., 2021) that collected 644 fecal samples from 48 preterm infants (including 14 with NEC) for mNGS found that children with NEC had reduced gut microbiome diversity. The relative abundance of *Bifidobacterium longum* was significantly reduced, whereas the relative abundance of *Enterobacter cloacae* was significantly increased, and the composition of the microbiome prior to the onset of disease was significantly different from that of controls. This severe dysregulation of microbiome diversity may lead to intestinal inflammation and barrier function disruption (Wang et al., 2009). This reduced microbial diversity aligns with current understanding: the underdeveloped gut of premature infants – particularly those with extremely low birth weight – lacks the anaerobic environment required to sustain *obligate anaerobes (* La Rosa et al., 2014). Consequently, *Enterobacteriaceae* proliferate dominantly (Warner et al., 2016). While mNGS provides mechanistic insights into this dysbiosis, it offers limited near-term clinical utility given the fundamental physiological constraints of extremely low birth weight infant gut development (Stewart et al., 2016). In addition, strain specificity is key to accurate identification of the cause of disease. It has been suggested that different strains within the same species may have completely opposite biological effects, like non-toxin-producing strains of *Clostridium acetobacter* can be used as probiotics, while toxin-producing strains, like β-hemolysin and neuraminidase genes, are important pathogens of diseases such as NEC, which can damage the intestinal mucosa. Therefore, the organic combination of traditional culture methods and mNGS is necessary to fully assess the diversity of the intestinal flora and identify pathogenic strains (Cassir et al., 2016). Some commensal bacteria can also affect the gut in several ways, such as prevention of apoptosis (Hooper et al., 2001), improvement of intestinal barrier function (Rakoff-Nahoum et al., 2004), establishment of intestinal mucus (Johansson et al., 2008), maturation of glycoconjugate patterns (Grizotte-Lake et al., 2018), improvement of immune responses, and maturation of intestinal vasculature system patterns (Stappenbeck et al., 2002). For instance, *Saccharomyces boulardii* (*S. boulardii*), a non-colonizing *Saccharomyces cerevisiae variant*, transiently persists in the neonatal gut as an early pioneering agent, Its cell wall *β* 111glucans and mannoproteins engage epithelial and dendritic cells (DCs) via pattern recognition receptors (*e.g.*, Dectin-1, TLR2), rapidly upregulating tight junction proteins (ZO-1, occluding) to restore and reinforce intestinal barrier integrity (Thomas et al., 2009), Concurrently, this yeast primed mucosal immunity by inducing DC maturation (CD80/CD86 upregulation) and stimulating cytokine secretion (IL-1*β*, IL-12,TNF-*α*, IL-10) (Rodrigues et al., 2000). Furthermore, *S.boulardii* significantly elevates secretory IgA (slgA) production, strengthening immunological defenses (Czerucka et al., 2000).A prospective study of very low birth weight infants using shotgun mNGS found that the use of *Bifidobacterium longum NCDO 2203* was associated with a significant reduction in microbiome-associated antimicrobial resistance compared to supplementation with the *probiotic Lactobacillus rhamnoses LCR35* or no supplementation, and that the beneficial effect was associated with the concomitant feeding of breast milk oligosaccharide (Neumann et al., 2023). A study collecting fecal samples from 113 preterm infants aged 24–32 weeks at 21 days postnatally found that the abundance of *bifidobacteria*, specifically *Bifidobacterium breve*, was significantly associated with low intestinal permeability. *B. breve* was more abundant in breastfed preterm infants and was associated with the maturation of the intestinal barrier. It has a highly specialized genetic ability to break down oligosaccharides from human milk and host-derived glycoproteins. These genetic features allow *B. breve* to effectively colonize the intestines of preterm infants and promote the maturation of the intestinal barrier. Therefore, the results provide a new strategy for the prevention and treatment of leaky gut in preterm infants by supplementing with specific *Bifidobacterium strains* and HMOs to promote the maturation of the intestinal barrier, thereby reducing the occurrence of complications such as NEC. Clinical modification of gut microbiota composition is an option to combat NEC, which is important for improving the health and survival of preterm infants (Morgan et al., 2020).

In summary, mNGS plays an important role in studying the relationship between neonatal gut injury and the gut microbiome. Using mNGS, researchers have been able to identify compositional and functional changes in the neonatal gut microbiome that are associated with the development of neonatal gut injury. Future studies could further use mNGS to explore the dynamics of the neonatal gut microbiome and develop new diagnostic and therapeutic strategies.

### 4.2 Neonatal infectious diseases

#### 4.2.1 Neonatal sepsis

Sepsis is a common condition in neonatal intensive care units. Conventional culture methods demonstrate limited efficacy in pathogen detection for suspected infections: a majority of samples (>50%) fail to yield potential pathogens indicating substantial presence of non-cultivable organisms, These necessitates molecular techniques to establish definitive etiology (Wang et al., 2020). mNGS has the advantage of rapidly and simultaneously detecting more gene sequences of suspected pathogens and is unaffected by antimicrobial drugs, which can provide a reference for severe neonatal infections that are difficult to diagnose or poorly detected by traditional testing techniques. In terms of diagnostic positivity, a retrospective study in children (neonatal sample size of 5.9%) found that mNGS was approximately 45.0% more sensitive than conventional microbiological tests (CMT) in distinguishing whether the disease was infectious (75.0% *vs.* 30.0%, *P*< 0.001) and approximately 60% more sensitive than CMT in distinguishing whether the disease was fungal (93% *vs.* 43.7%, *P*< 0.001) (Tao et al., 2022). A retrospective study in children found that in patients with sepsis under 6 years of age or in an immunosuppressed state, mNGS had a higher positive rate (75.3%) and a comparable negative rate (75.0%) compared to CMT (Zhu et al., 2023). To counter the effect of antibiotics, a comparison of blood cultures and mNGS in preterm infants with sepsis (*n*=45) showed that the rate of positive blood mNGS tests for pathogens was higher than the rate of positive blood cultures and was unaffected by the duration of antibiotics (40% *vs.* 3%, *P*<0.001) *(* Lou et al., 2024). A retrospective study comparing sepsis in preterm infants treated with antibiotics found a higher rate of positive pathogen detection by mNGS than by blood culture, regardless of whether antibiotics had been administered for more than 10 days (Lou et al., 2024). In terms of targeting specific pathogenic infections, a case report of neonatal sepsis demonstrates the value of mNGS in the etiological diagnosis of severe neonatal infections, such as the identification of human parainfluenza virus (Chen et al., 2023). Therefore, mNGS can be considered for identifying putative pathogens when there is a high clinical suspicion of infection, and the pathogen cannot be detected by blood culture.

#### 4.2.2 Infection-associated neonatal pneumonia

Infection-associated neonatal pneumonia is a leading cause of neonatal mortality, and timely and accurate identification of the pathogen is essential to improve survival in critically ill patients, where delayed or inadequate antimicrobial therapy can lead to poor outcomes. For example, mNGS has a higher positive pathogen detection rate in clinical samples of lower respiratory tract infections than conventional pathogen testing (91.70% *vs.* 37.60%), and its rapid detection time and reduced sensitivity to the effects of antimicrobial drug use make mNGS a valuable adjunct to diagnostic and therapeutic decision-making for suspected lower respiratory tract infections in clinical settings (Lv et al., 2024). In a multiple case report (West et al., 2022), mNGS was applied to a neonate with pneumonia without specific clinical symptoms, and the pathogenic Legionella pneumophila was found (sequence number 24055, relative abundance 96.18%), which was cured and discharged from hospital with timely administration of medication (Liu et al., 2024). The mNGS is also helping to improve clinical outcomes in preterm infants by supporting clinical outcome monitoring and easy adjustment of treatment regimens. For example, a retrospective study comparing mNGS and routine microbiological testing of bronchoalveolar lavage fluid in neonates (n=18) with lung infections supported by ECMO found a higher rate of pathogen positivity by mNGS than by CMT (77.8% *vs.* 44.4%, *P*=0.04), with nine of these children undergoing therapeutic changes based on mNGS results. A controlled study of infectious pneumonia in preterm infants found that mNGS-assisted clinical management reduced antibiotic duration, frequency of adjustments, length of hospital stay, improved the efficiency of clinical diagnosis of infection-associated pneumonia in preterm infants, and improved pathogen detection and clinical outcomes (Wang et al., 2022). Therefore, mNGS can detect pathogens earlier and more sensitively than CMT and may play an important role in detecting neonatal pneumonia pathogens and optimizing antibiotic therapy.

#### 4.2.3 Infection-associated neonatal meningitis

Neonatal infection-associated meningitis is most caused by bacteria or viruses and has an atypical clinical presentation. Early diagnosis relies on CSF culture, but the positive rate is low. The use of mNGS to assist in the diagnosis of pathogens may help in early etiological diagnosis. For example, several (Zhan et al., 2021) cases of meningitis in neonates with *Mycoplasma* infection were diagnosed by mNGS and the treatment plan was determined based on the sequencing results (Che et al., 2023). A retrospective, single-center, case-control study of infectious meningitis in newborns (*n*=69) found that mNGS had a higher diagnostic accuracy than CSF cultures (*PPV* 100%, *NPV* 38.10%, *concordance* 62.32%, *AUC* 0.750, 95% *CI* 0.636-0.864), and the difference was statistically significant in accelerating the diagnostic rate and adjusting anti-infective medication (Zou et al., 2024). A prospective study in neonates with suspected central nervous system infections (*n*=88) comparing the diagnostic accuracy of mNGS, conventional methods and mNGS combined with Whole Exome Sequencing found that mNGS was more accurate than conventional methods (27% *vs.* 6.3%, *P*=0.002) and that the combination of mNGS and Whole Exome Sequencing may significantly improve a etiological diagnosis and effectively guide clinical strategies (Ge et al., 2021). Therefore, mNGS has the potential to reduce the overall cost and burden of disease management of neonatal infections when all conventional methods fail to identify the culprit (Zhang et al., 2022).

#### 4.2.4 Congenital tuberculosis

According to a 2018 World Health Organization report, around 10,000 newborns worldwide are infected with *Mycobacterium tuberculosis*, with women and children under the age of 15 accounting for 32% and 11% of tuberculosis cases, respectively (W. Global tuberculosis report, 2019). Congenital tuberculosis often develops 2–4 weeks after birth, the clinical manifestation lacks specificity and early diagnosis is not easy, the disease progresses rapidly, the mortality is as high as 20-50% and the prognosis is poor, early diagnosis can significantly improve the prognosis (Du et al., 2021). However, *Mycobacterium tuberculosis* has a long routine culture cycle, a high negative rate, and the immature immune system of neonates makes detection difficult, so new detection techniques are needed. A case report showed that a child was admitted to hospital with cough, fever and dyspnea on the 20th day after birth, and sputum culture, blood culture, CSF culture, GM test and bone marrow examination were completed without any abnormalities, and Mycobacterium tuberculosis infection was detected by mNGS. And he was discharged from hospital after anti-tuberculosis treatment, with no recurrence in 2 years of follow-up (Fu Fenfen et al., 2024).Another meta-analysis (*n*=693), which included 8 studies, found that mNGS had a sensitivity of 62% (95% *CI* 0.46-0.76) and a specificity of 99% for diagnosing tuberculous meningitis, so it is specific for diagnosing tuberculous disease, but with moderate sensitivity. There is, thus,a need to develop more sensitive tests to aid diagnosis (Xiang et al., 2023).

In summary, mNGS offers distinct advantages for severe neonatal infections: it rapidly detects broad pathogen gene spectra simultaneously, remains unaffected by prior antimicrobial exposure, and addresses limitations of conventional diagnostics in complex cases. Low microbial biomass samples (e.g., blood) are prone to false-positive results due to amplification of cell-free microbial DNA translocated across mucosal barriers (Blauwkamp et al., 2019). Nevertheless, sequencing data from such specimens may still inform clinical interventions in suspected infectious diseases (Salter et al., 2014).

### 4.3 Neonatal jaundice

Neonatal jaundice is a common condition that occurs mostly in the first week of life, with a prevalence of up to 80 per cent in preterm infants (Rennie et al., 2010). Studies suggest that neonatal jaundice may be associated with a number of long-term health problems, including childhood asthma (Huang et al., 2013), type 1 diabetes (McNamee et al., 2012) and visual impairment (Hou et al., 2014). Therefore, the prevention and treatment of neonatal jaundice has become a clinical concern. mNGS revealed significantly increased gut microbial diversity in cholestatic jaundice patients, marked by elevated *Bifidobacterium* spp. abundance and enrichment of pathogenic invasion-associated functional genes. Conversely, in breast-milk jaundice, the abundance of *Bifidobacterium* spp. Is markedly diminished. Critically, nine *Bifidobacterium* species and galactose metabolism-related functional genes demonstrated inverse correlations with serum direct bilirubin concentrations (Zhou et al., 2019). This study demonstrated the relationship between gut flora and jaundice, suggesting that modulation of gut flora like supplementation with *Bifidobacterium bifidum*, may be potentially beneficial in the treatment of jaundice. However, the mean age of the population included in this study was in early childhood and is underrepresented in the relationship between neonatal jaundice and gut flora. In another study, 25 infants (6 with neonatal jaundice and 19 without neonatal jaundice) were enrolled and fecal samples were collected at postnatal months 0, 1, 3, 6 and 12 for mNGS, which showed that the diversity of fecal microorganisms in patients with neonatal jaundice was significantly lower than that in patients without neonatal jaundice with a significant decrease in the abundance of *Gemella* spp. in particular. Significant increases in some microbial functions such as propionyl-CoA acyl group transferase and 3-oxoacyl carrier protein synthetase I was found, whereas other functions like fructose-1,6-bisphosphatase III) were significantly reduced. After recovery from treatment, there were still significant differences in the microbial communities of the two groups at 1 month, but these differences diminished over time. This study uses a new technique to show that neonatal jaundice is associated with changes in the composition and function of the gut flora, and that early intervention in the gut flora may be helpful in the management of neonatal jaundice. This technique holds promise for the future search for additional biomarkers associated with jaundice (Ding et al., 2021). However, the small sample size of this study limits the generalizability of the findings and should be expanded in the future. Future studies can further use the mNGS to explore the dynamic changes in the intestinal flora of children with neonatal jaundice, reveal the specific role mechanisms of different flora in the development of jaundice, and then develop new diagnostic and therapeutic strategies based on the intestinal flora, providing new ideas and methods for the clinical management of neonatal jaundice.

Current studies exhibit four key limitations: systematic omission of RNA sequencing compromises viral pathogen coverage; clinically indicated enrollment introduces selection bias; short follow-up obscures long-term outcomes; and multifactorial confounders in early microbiota interventions impede causal attribution. We propose establishing multicenter, large-scale cohorts with extended follow-up to validate mNGS clinical utility in neonatal diseases.

## 5 Discussion

Normally, the fetus develops in a sterile uterus. However, due to a variety of factors, preterm infants must continue to develop in the neonatal intensive care unit for part of the postnatal period, and this environmental change results in the infants being prematurely colonized by microorganisms during critical developmental transitions and affecting the infant’s immune system (Claud et al., 2013). Timely and accurate etiological diagnosis is a prerequisite for rational treatment. mNGS not only detects pathogens that are difficult to detect by traditional methods, but also allows portable Nanopore sequencing-based devices to deliver results within 6 hours, such as the successful identification of pathogens responsible for neonatal infection-related deaths in post-mortem blood and tissue samples (Baillie et al., 2023).In addition, mNGS can reveal the central role of the neonatal microbiome in disease development. It was found that early differences in the gut microbiota between neonates with prevalent heart disease and normal neonates may be correlated with oxygen levels. Neonates with CHD had a significant effect on the relative abundance of the gut core microbiota (*P*=0.04), which the method of oxygen delivery may affect the stability of the microbiota (Renk et al., 2024). In the Free State of South Africa, the use of mNGS to analyze fecal samples collected from a longitudinal cohort of 17 asymptomatic newborns revealed the predominance of *enteroviruses B and C* (Mogotsi et al., 2024). These findings could help investigate strategies for the control and treatment of infections.

Compared to conventional 16S rRNA sequencing [**Table 4** (Clinical Microbiology Group of Chinese Society of Laboratory Medicine CMGoCSoMaI, Society of Clinical Microbiology and Infection of China International Exchange and Promotion Association for Medical and Healthcare WH, [[NoYear]])], mNGS has a higher resolution and is able to reach the species or even strain level. For example, 16S rRNA sequencing can only identify to genus level, whereas mNGS can distinguish between different species within the same genus such as Escherichia coli *(E. coli*) and *Shigella*. In addition, macro-genome sequencing can analyze not only bacteria, but also viruses, fungi and parasites, providing more comprehensive information on microbial communities and resolving microbial functional genes like antibiotic resistance genes, virulence factors, metabolic pathways and so on. For example, in the study of neonatal sepsis, mNGS not only identifies the pathogen but also predicts its drug resistance, providing an important reference for clinical management (Pammi et al., 2020). A study applying mNGS to stool samples collected from 144 preterm (<30 weeks gestational age) and 22 term infants from 3 to 22 days postnatal found that *E. coli strains* associated with the risk of NEC have a specific genetic signature that is linked to iron acquisition, the phosphotransferase system, and D-serine metabolism; and that preterm infants carrying urinary pathogenic *E. coli* are at significantly increased risk. Urinary pathogenic *E. coli* is not only associated with the development of NEC, but also with NEC-associated mortality, and these findings point the way to further exploration of the epidemiological characteristics of UPEC and how to reduce the risk of NEC through early identification and intervention in the future (Ward et al., 2016). Other studies have applied shotgun mNGS to a set of twin faecal samples (one suffering from NEC, one not) and found that there was a clear separation in the set of functional groups prior to the onset of NEC, focusing mainly on carbohydrate metabolism, which provides a possible direction of investigation for subsequent studies (Claud et al., 2013). For example, a deficiency of glyoxylate reductase may lead to elevated levels of glyoxylate, which inhibits the vitamin D receptor from interacting with vitamin D response elements (Patel et al., 1997). Whereas vitamin D has been shown to be involved in the pathogenesis of inflammatory bowel disease and to have immunomodulatory effects (Lim et al., 2005). A study found that *E. coli* in the intestines of preterm infants in the NEC group exhibited higher transcription ratios of hypoxia-related genes and outer membrane protein genes, suggesting that a low-oxygen, high osmolality environment may exist in the intestines prior to the onset of NEC; and lower genes for nitrate detoxification enzymes, suggesting that the nitrate level is low, providing a new way of thinking for the study of the pathogenesis of NEC (Sher et al., 2020). mNGS has an important role in neonatal infection-associated diseases, altered intestinal flora, and inherited metabolic diseases. mNGS is useful for hypothesis testing, but making clinical decisions cannot always be safely made on such predictions.

**Table 4:** Comparison between mNGS and 16S rRNA sequencing (Clinical Microbiology Group of Chinese Society of Laboratory Medicine CMGoCSoMaI, Society of Clinical Microbiology and Infection of China International Exchange and Promotion Association for Medical and Healthcare WH, 2021).

Items	mNGS	16S rRNA sequencing
Principles	Total microbial DNA from environmental samples was mechanivally fragmented, ligated with universal adapters, PCR-amplified, and sequenced. Resulting short reads were then assembled into contgs for subsequent analysis	Amplicon sequencing targeting hypervariable regions (e.g., V3-V40 of the 16S rRNA gene, following PCR amplification with universal primers
Scope	This approach provids comprehensive coverage of microbial genomes within the sample, encompassing bacteria, fungi, viruses, and other microbial domains	mainly targeting bacteria and archaea
Species identification	Ability to identify microorganisms down to the species or even strain level	Often only identified to genus or species level, and in some cases with limited accuracy at species level
Volume of data and complexity of analysis	Large volume of data and complex bioinformatics analyses	Relatively small amount of data and simple to analyse
Cost and time	Higher cost and longer time	Lower cost and shorter time
Fields of application	Applicable for comprehensive profiling of microbial community composition and function, including environmental microbiology and clinical diagnostics	Routinely employed for composition and diversity analysis of microbial communities in fields such as microbial ecology and environmental science

However, there are still many challenges to the application of mNGS in the neonatal setting. Neonatal sample sizes are typically small and have a high percentage of host DNA, which can affect the depth of sequencing and analysis of microbial DNA. To overcome this problem, researchers have developed a variety of techniques to remove host DNA, such as selective lysis of host cells and digestion of host DNA using host DNA enzymes, as well as optimized sample processing methods like microbial DNA enrichment, and library construction methods such as whole genome amplification. In addition, the resulting data volumes are huge, typically tens of gigabytes. And data analysis involves multiple steps such as quality control, species annotation and functional analysis, requiring sophisticated bioinformatics tools and expertise. Interpretation of microbiome data needs to consider host factors such as age, diet and antibiotic use. And early interpretation of neonates as a group in particular places greater demands on researchers. Furthermore, unresolved challenges persist regarding cost-effectiveness and data privacy in neonatal genomics. Crucially, the current evidence base for mNGS across disease spectra lacks robust validation through large-scale prospective trials, Future multicenter prospective studies are imperative to address this critical evidence gap.

There have been several studies looking at how to improve the efficacy of mNGS. For example, in terms of sample type selection, a 2-year retrospective cohort study in pediatric settings found no significant increase in efficacy or antimicrobial duration when plasma was chosen as the sample for testing compared to blood samples. This study found that mNGS in plasma was more useful in patients with immunodeficiency, but less valuable than expected in patients with endocarditis (Lee et al., 2020). A study of *Klebsiella* pneumoniae, a common clinical bacterium, found that owing to mNGS identification is based on microorganism’s sequence rather than its reads number, blood mNGS and secretion mNGS were comparable in terms of specificity, but blood samples were more effective in critically ill patients. In addition, the advantages and disadvantages of different sample sources varied, with plasma samples having an advantage in the identification of viruses and a disadvantage in the detection of bacteria and fungi compared with alveolar lavage samples (Yao et al., 2023). In terms of high-throughput sequencing and timing of sample collection, a study that recruited 168 neonatal patients with suspected infection and collected blood samples (*n*=153), cerebrospinal fluid samples (*n*=127) and respiratory samples (*n*=51) for mNGS found that it is optimal to use cfDNA and RNA to assess the performance of mNGS and the optimal timing for neonatal mNGS testing is between the start of continuous antimicrobial therapy 1 to 3 days (Chen et al., 2022). There are fewer studies related to the improvement of mNGS, and more samples and multidisciplinary collaboration are needed to address related issues.

## 6 Conclusion

mNGS has shown promising applications in the early diagnosis of neonatal severe infections, individualized treatment guidance, and inherited metabolic diseases. Although cost-effectiveness and standardization still need to be explored, with the accumulation of technology iteration and clinical validation, mNGS is expected to become a core tool for the early diagnosis, precise treatment, and prognosis assessment of neonatal severe infections, with considerable potential for clinical application in the long term.
